# The evolution and future directions of pressure injury wound dressing research: a bibliometric analysis (2005–2025)

**DOI:** 10.1097/MD.0000000000048458

**Published:** 2026-04-24

**Authors:** Chenxin Zhang, Yi Liu, Xian Ma, Liping Zhang, Zhenghui Dong

**Affiliations:** a School of Nursing, Xinjiang Medical University, Xinjiang, Urumqi, China; b The Sixth Affiliated Hospital of Xinjiang Medical University, Xinjiang, Urumqi, China; c Health Care Research Center for Xinjiang Regional population, Xinjiang, Urumqi, China.

**Keywords:** bibliometrics, dressings, knowledge mapping, pressure injury, wound care

## Abstract

**Background:**

This research investigates advancements in “dressings for pressure injuries (PIs)” by analyzing literature from the Web of Science Core Collection spanning 2005 to 2025. The study aims to systematically map research trends in this field through bibliometric approaches.

**Methods:**

A bibliometric analysis was conducted using VOSviewer and CiteSpace to examine author collaborations, geographical distributions, keyword co-occurrence networks, and temporal evolution of research hotspots.

**Results:**

The evolutionary trajectory showed distinct phases: early studies (2005–2015) prioritized material characterization, while recent advances (2016–2025) focused on translating bioactive dressings into practice and integrating multidisciplinary approaches.

**Conclusion:**

Future efforts should prioritize bridging the innovation-clinical translation gap of smart wound dressings, deepening the micro-mechanistic exploration of dressing-wound microenvironment interactions, and strengthening cross-cluster knowledge integration across basic research, clinical practice, and interdisciplinary fields. These directions will drive the advancement of precision, intelligence, and standardization in pressure injury dressing care.

## 1. Introduction

### 1.1. Global challenges of PIs in healthcare

Pressure injuries (PIs), a pervasive, often underestimated healthcare concern, affect patients of all ages in a variety of care settings.^[1]^ PIs, an ischemic-hypoxic lesion caused by impaired blood circulation due to localized pressure, friction, or shear forces, is commonly observed in individuals with prolonged bed rest, impaired mobility, edema, or incontinence.^[2]^ The aging global population and rising prevalence of chronic diseases have established PIs as a significant challenge for healthcare systems worldwide. Elderly populations exhibit heightened vulnerability due to age-related skin changes, including epidermal thinning, reduced collagen density, xerosis, and diminished elasticity,^[3]^ Moreover, comorbidities such as mobility limitations and incontinence further elevate their risk, making this demographic the most frequent hospital-acquired pressure ulcer (PU) cases.^[4]^ Hospitalized patients face additional predisposing factors including prolonged immobility, malnutrition, tissue edema, and inadequate repositioning practices.^[5]^ Globally, PU prevalence reaches 20%, with intensive care units, geriatrics, and internal medicine departments demonstrating the highest incidence rates.^[6]^ Consequently, PU occurrence has emerged as a critical indicator for evaluating patient safety standards in clinical settings.^[7,8]^ The COVID-19 pandemic further emphasized the urgency of this issue, as healthcare workers experienced medical device-related facial PIs due to prolonged use of personal protective equipment. This highlights the need for prevention and management strategies to extend beyond patient populations to include healthcare providers.

### 1.2. Clinical value of advanced dressing research

The National Pressure Injury Advisory Panel consensus emphasizes that most PIs are preventable, with preventive measures demonstrating greater cost-effectiveness compared to treatment expenditures. Consequently, targeted preventive strategies play a pivotal role in PI management.^[9]^ Among preventive modalities, prophylactic dressings have gained widespread clinical acceptance due to their ease of application, patient comfort enhancement, and demonstrated efficacy in risk mitigation. The 2014 international guidelines for PI prevention explicitly recommend incorporating preventive dressings into standard care protocols.^[10]^ Multiple studies have validated the effectiveness of dressings in reducing PI incidence.^[11,12]^ These dressings function by dual mechanisms: shielding vulnerable skin surfaces from shear forces and friction, primary contributors to PI pathogenesis; and redistributing mechanical pressure to maintain dermal microcirculatory integrity. Current clinical practice employs various advanced dressing materials for PI prevention, including polyurethane films, hydrocolloids, foam-based composites, hydrogels, and liquid-applied barrier films.^[13]^

Hydrocolloid dressing, categorized as an active hydrophilic wound care material, is synthesized through the integration of hydrophilic polymer particles with rubber elastomers.^[14]^ Its semipermeable polyurethane outer layer containing sodium carboxymethyl cellulose exhibits robust autolytic debridement capabilities while maintaining a localized moist microenvironment through exudate absorption.^[15]^ This dressing enhances tissue oxygenation and microcirculation through its oxygen-permeable yet waterproof structure, making it clinically valuable for pressure injury prevention and management.^[16]^ Foam dressings demonstrate comparable clinical efficacy through distinct mechanisms. Characterized by a soft porous matrix, these dressings establish microbial barriers while regulating moisture and thermal retention, thereby optimizing cutaneous conditions for pressure ulcer prophylaxis.^[17]^ Silver-impregnated antimicrobial dressings provide broad-spectrum bactericidal activity via multiple mechanisms, including ion release and membrane disruption. Exhibiting low irritancy and minimal resistance development, they are particularly suitable for managing infected chronic wounds like diabetic ulcers and burns.^[18]^ Comparative studies reveal novel dressings’ superiority over traditional gauze in maintaining physiologic wound temperature (approximating 37°C), which enhances mitotic activity and reduces infection risks associated with frequent dressing changes.^[19]^ Yan et al^[20]^ demonstrated equivalent effectiveness between hydrocolloid and foam dressings in preventing nasal bridge PIs among elderly noninvasive ventilation patients, both outperforming cotton padding. In neonatal care, Chen team^[21]^ observed significantly shortened mechanical ventilation duration and hospital stays when applying hydrocolloid dressings for nasal septum protection during respiratory support.

The dressings can form a nano-ionic layer at the wound interface, effectively minimizing adhesion between the wound surface and dressing material, thereby reducing tissue trauma during dressing replacement. Furthermore, these dressings provide a physical barrier that isolates the wound from external microorganisms, preventing pathogenic invasion and enhancing wound protection. This mechanism supports healing progression, accelerates clinical recovery, and ultimately improves patients’ quality of life.^[22]^ Consequently, given that PIs exhibit distinct characteristics at different stages, and considering the challenges of treatment (which involve prolonged duration, high costs, and limited consensus on the efficacy of various dressings) selecting stage-specific dressings not only optimizes therapeutic outcomes and nursing efficiency in pressure injury management but also aligns with the core clinical need for reliable treatment protocols and optimized dressing selection.^[23,24]^

### 1.3. Necessity and innovation of bibliometric analysis

While meta-analyses and randomized controlled trials have examined the effectiveness of certain dressings, a comprehensive macroscopic analysis of research hotspots, knowledge evolution trajectories, and international collaboration patterns in this field is still absent. Bibliometric methods enable a quantitative analysis of 2 decades of academic output trends, core author networks, and critical technological inflection points, thereby identifying underexplored research directions. This research applies bibliometric and visualization analyses to explore the current situation and evolutionary trends of studies on dressings for PIs in the past 20 years, aiming to provide actionable insights for advancing clinical practices and guiding future research efforts.

## 2. Research methods and data sources

### 2.1. Research methods

Bibliometrics, which originated in the first part of the 20th century and evolved into an independent discipline by 1969,^[25]^ is now extensively applied in literature analysis.^[26]^ This method offers a quantitative approach to review and investigate existing literature within specific domains.^[27]^ By extracting detailed information such as authors, keywords, journals, countries, institutions, and references, bibliometric analysis enables the systematic characterization of disciplinary development.^[28]^ Modern computational techniques further enhance this approach by generating graphical and visual outputs. Co-citation, a widely used bibliometric technique, identifies relationships between 2 documents cited simultaneously by 1 or more publications. As emphasized by Ma and Xi,^[29]^ co-citation visualization facilitates data interpretation, enhances comprehensiveness, and reveals intrinsic connections among information.

Knowledge mapping is essential for structuring domain knowledge and uncovering research trajectories. Two complementary software tools, CiteSpace (Drexel University, Philadelphia) and VOSviewer (Centre for Science and Technology Studies, Leiden University, Leiden, The Netherlands), are widely employed for this purpose. CiteSpace applies data normalization grounded in set theory to assess the similarity among knowledge units. Through similarity algorithms, it generates timezone and timeline views based on temporal slices, enabling researchers to observe knowledge evolution, historical spans of literature clusters, and future trends.^[30]^ In contrast, VOSviewer adopts probability theory-based normalization, offering diverse visualizations such as network views (depicting inter-element relationships), overlay views (analyzing topic coverage), and density views (highlighting hotspot distributions). Renowned for its user-friendly interface and visually appealing outputs, VOSviewer is a preferred tool among researchers.^[31]^ This study integrates both tools to explore bibliometric methodologies, leveraging their complementary strengths to advance research insights.

### 2.2. Data sources

The Web of Science Core Collection (SCI-EXPANDED) was employed by this study as the primary data source, as this index covers high-quality academic journals worldwide to ensure the comprehensiveness and accuracy of retrieved data. The search strategy combined the following terms: topic search = (“pressure injury” or “pressure ulcer” or “pressure sores” or “decubitus ulcer” or “decubitus bedsores”) and topic search = (“dressings” or “wound dressings”), covering the period from January 2005 to February 2025 (retrieval date: February 5, 2025).

Explicit inclusion and exclusion criteria for literature were established as follows:

Inclusion criteria: literature types were restricted to peer-reviewed journal articles (Articles) and systematic reviews/meta-analyses (Review Articles); early access articles were included only if they had completed peer review, been officially accepted by the journal, and clearly addressed the application of dressings in PIs in their full text or detailed abstract; publications were limited to English, as the research team’s language proficiency is primarily in English and the SCI-EXPANDED index mainly covers high-quality English journals, which can fully reflect international research frontiers; research content focused on the application, efficacy evaluation, and mechanism exploration of dressings for PIs.

Exclusion criteria: conference papers, which were excluded because most are preliminary research reports with incomplete data, unvalidated conclusions, and lack strict peer review compared to journal articles; “In press” or “under review” manuscripts that had not been officially accepted to avoid including unconfirmed research results; non-English publications to prevent information bias caused by language barriers in data extraction; nonsystematic review articles (other than the included Review Articles) due to the lack of original research data; irrelevant publications that only mentioned PIs or dressings but did not address the application of dressings in PIs.

**Figure 1.**
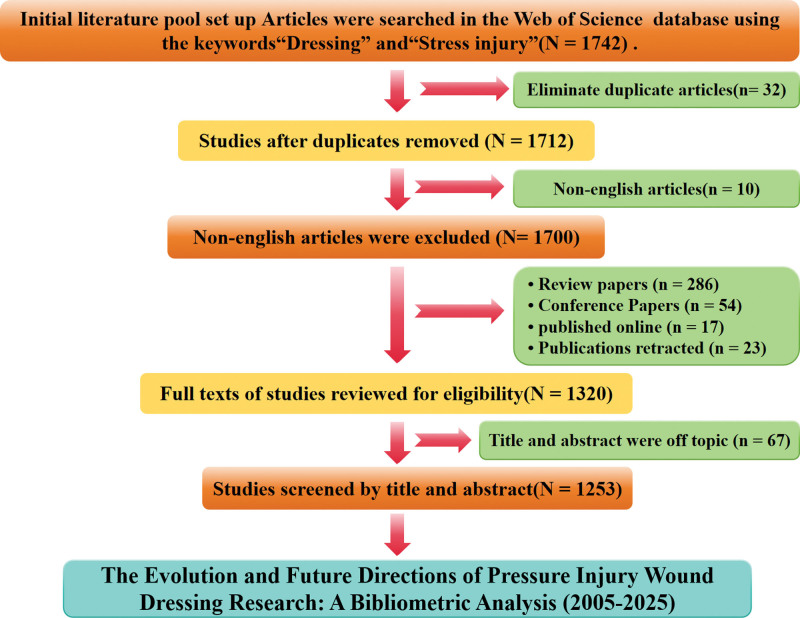
Article selection process for bibliometric mapping analysis and systematic review. N = Total number of articles, n = number of articles removed.

Initial searches yielded 1742 records; after deduplication (32 removed via EndNote) and exclusion of non-English publications (10 removed), 1700 English journal articles remained. Next, full-text eligibility review (conducted by 2 independent researchers, with third-researcher consultation for disagreements) excluded 380 records: 286 reviews (excluded as this bibliometric analysis focused on original research progress of pressure injury dressings), 54 conference papers (excluded for being preliminary and less standardized than peer-reviewed journal articles), 17 early access manuscripts (excluded as they were unfinalized and subject to revision), and 23 retracted publications (excluded due to unreliability from academic issues or errors), leaving 1320 studies. Subsequent title/abstract screening (same dual-researcher + third-researcher consultation process) excluded 67 records, these focused on nonpressure injury dressings or nonclinical “stress injury,” which were inconsistent with the theme, ultimately retaining 1253 articles for content analysis and bibliometric mapping (Fig. 1).

## 3. Descriptive statistics

The analysis of 1253 articles in this study revealed contributions from 6128 authors affiliated with 2218 organizations across 74 countries. The articles were featured in 375 journals and cited 28,136 references from 7668 journals.

**Figure 2.**
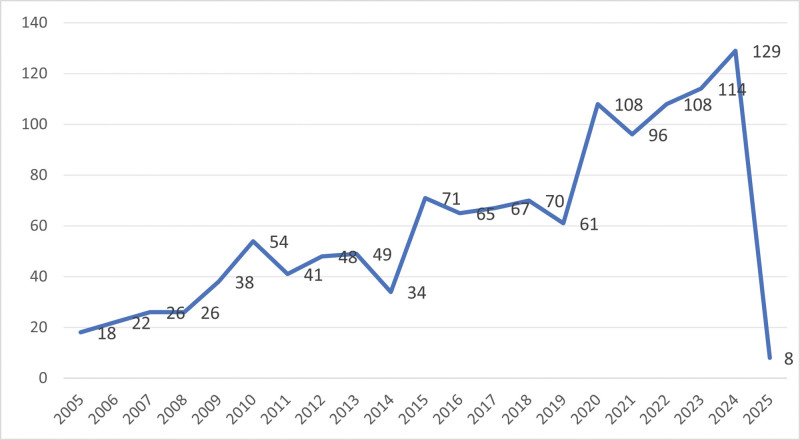
Illustrates how publications are spread from 2005 to 2025 in this field.

Figure 2 presents the chronological distribution of publications related to dressings for PIs. Collectively, research in this field has shown remarkable expansion, particularly after 2015. Annual publication volumes consistently exceeded 60 articles from 2015 to 2019, followed by a surge in 2020, with sustained output exceeding 90 articles annually. This indicates the increasing research interest and emerging significance of this field within the medical research domain.

### 3.1. Bibliometric analysis of authors

**Figure 3.**
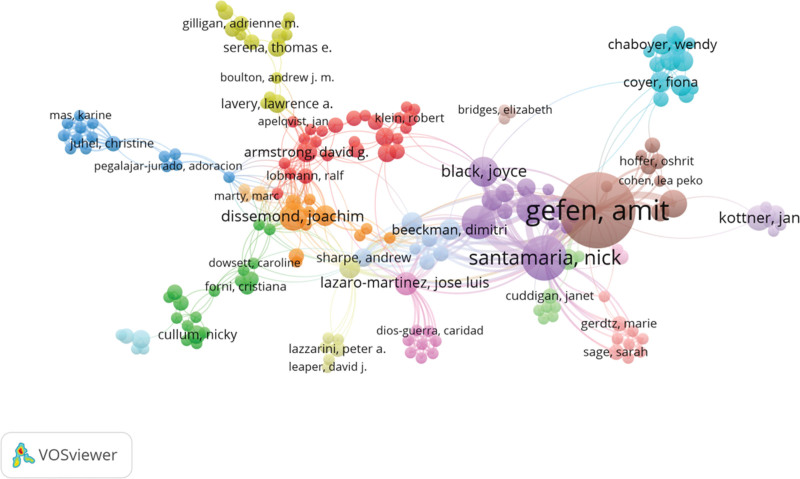
Authors’ bibliometric analysis.

Examining highly productive authors in pressure injury dressing research offers valuable insights into academic contributions and research trajectories. As illustrated in Table 1 and Figure 3, in this field, Gefen is the author with the highest productivity, with 42 high-quality publications. These works, known for their rigor and innovation, have received 773 total citations, averaging 18 citations per article. This not only reflects Gefen quantitative leadership but also emphasizes the foundational impact of their research on scholarly discourse and knowledge dissemination. Santamaria, with fewer publications (18 articles), achieves remarkable academic influence, accumulating 598 total citations and averaging 33 citations per article. This higher citation-per-article ratio indicates that Santamaria work addresses critical issues in the field, fostering significant academic dialogue and cementing their contributions as essential to the intellectual landscape of pressure injury dressing research. While Gefen extensive output establishes a broad knowledge base, Santamaria high-impact studies drive innovation and theoretical advancement. Together, their complementary efforts significantly propel the field forward.

**Table 1:** Top 10 authors in the field of pressure wound dressing.

Rank	Author	Documents	Citations	Average Citation/Publication
1	Gefen, amit	42	773	18.4
2	santamaria, nick	18	598	33.22
3	alves, paulo	11	300	27.27
4	black, Joyce	9	319	35.44
5	call, Evan	9	283	31.44

### 3.2. Bibliometric analysis of journals

**Figure 4.**
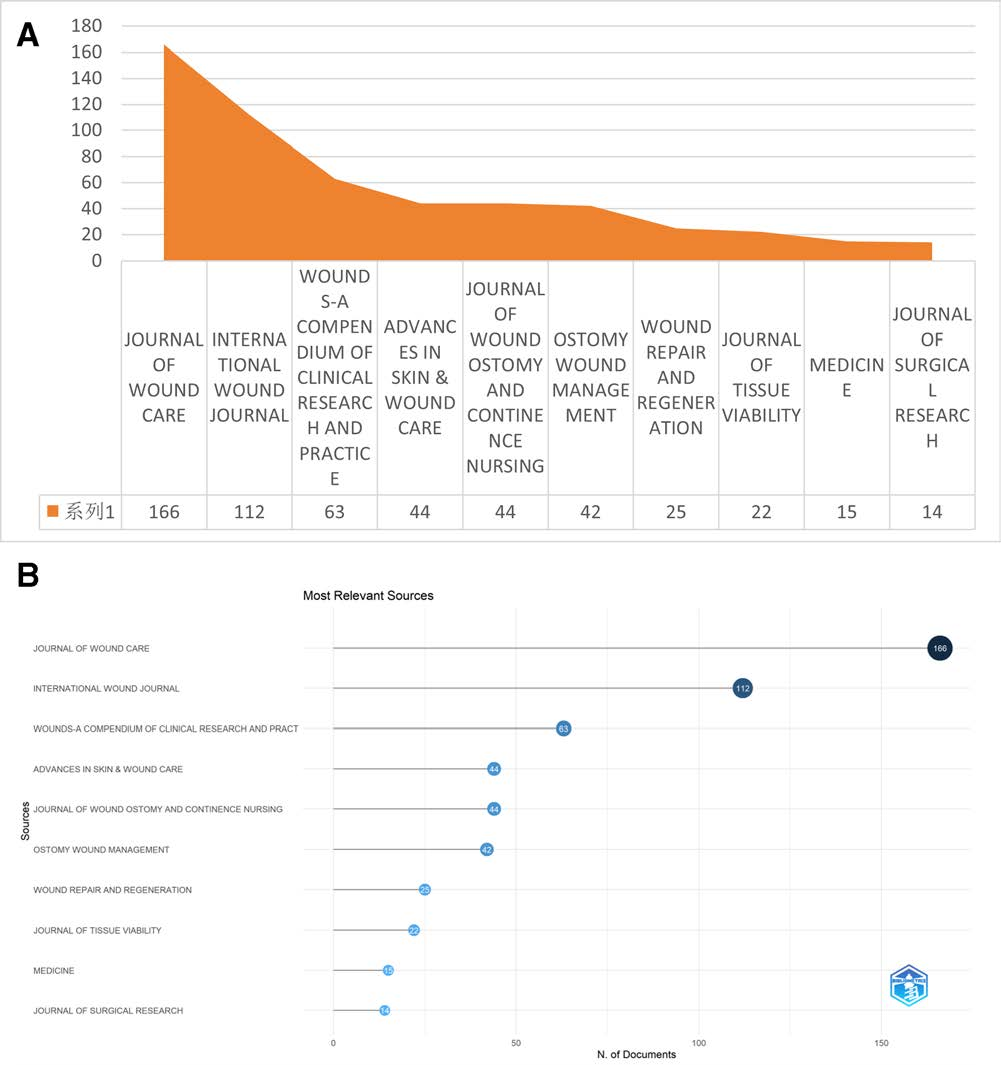
(A) Data overview, (B) visualization. N = number of documents.

In this study, a thorough and detailed statistical analysis was executed on the journals where the literature was published. The research spans nearly 2 decades, during which the investigation of journals publishing papers in this field revealed that, except for a very small number of general journals, the vast majority of papers were published in specialized journals in the fields of biotechnology and medicine. These specialized journals focus on research in specific areas and provide an important platform for academic exchange and the dissemination of research findings. Table 2、Figure 4A and Figure 4B detail the leading 10 journals based on publication volume, These journals are crucial for spreading academic research in this field.

**Table 2:** Top 10 journals in the field of pressure wound dressing.

Rank	Source	Publications	Citations	Average Citation/ Publication
1	Journal of Wound Care	166	1128	6.80
2	International Wound Journal	112	2656	23.71
3	Wounds: A Compendium of Clinical Research and Practice	63	572	9.08
4	Advances in Skin and Wound Care	44	457	10.39
5	Journal of Wound Ostomy and Continence Nursing	44	749	17.02
6	Ostomy Wound Management	42	943	22.45
7	Wound Repair and Regeneration	25	723	28.92
8	Journal of Tissue Viability	22	195	8.86
9	Medicine	15	100	6.67
10	Journal of Surgical Research	14	311	22.21

Among the numerous journals, Journals with particularly remarkable publication volumes are the Journal of Wound Care, International Wound Journal, and Wounds: A Compendium of Clinical Research and Practice. Specifically, the Journal of Wound Care published 166 articles, the International Wound Journal published 112 articles, and Wounds: A Compendium of Clinical Research and Practice published 63 articles. These high publication volumes reflect these journals’ sustained attention to and active promotion of research achievements in this field. Further in-depth analysis of citation metrics revealed that, among the relevant journals, the leading wound care journal is the one with the highest average citations per article. Wound Repair and Regeneration: this journal published 25 articles, each article has an average of 28.92 citations. Such a high average citation count fully demonstrates the superior quality of the articles published in this journal, which have garnered widespread attention and high recognition in research areas such as dressings and PIs. This indicates that the journal holds a significant position in academic research on wound care, and its published articles provide a rich reference base and innovative insights for subsequent studies.

A detailed analysis of the literature published in Wound Repair and Regeneration reveals that the journal primarily focuses on empirical research papers. Empirical research papers, based on actual observations and experimental data, exhibit strong scientific rigor and reliability, providing direct support for clinical practice and theoretical advancements in the field of wound care. Additionally, the journal consistently focuses on the field of wound care, covering all aspects of wound research, from prevention and diagnosis to treatment. It has developed a meticulously designed and focused platform tailored for both academic research and practical implementations in wound care.

### 3.3. Bibliometric analysis by country

**Figure 5.**
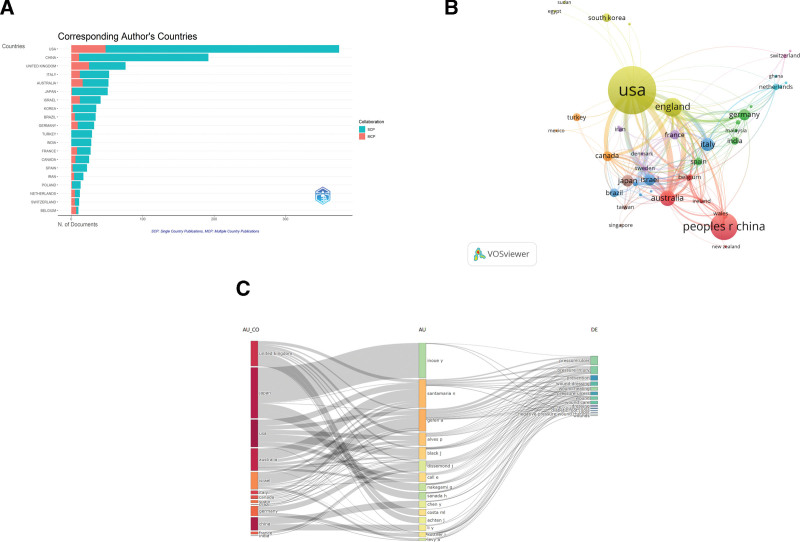
(A) Statistics of national publications, (B) The network map of collaboration between countries/regions was analyzed by VOSviewer, (C) 3-field plot on biomarkers (left field: countries; middle field: authors; right field: keywords). AU = author, CO = country, DE = keyword, MCP = multiple country publications, N = number of documents, SCP = single country publications, USA = United States of America.

In the research field of dressings and PIs, understanding the contributions of different countries is crucial for grasping the global research landscape. This research undertook a thorough and systematic examination of the publication contributions from the leading 35 countries in this domain. The analysis began with data visualization utilizing VOSviewer and Bibliometric software, and the corresponding visual outputs are displayed in Figure 5.

In Figure 5A, the vertical axis represents countries, while the horizontal axis shows the number of publications. Light green denotes publications independently authored by a single country, and red indicates publications resulting from multinational collaborations. The United States has the longest bar, representing the country with the highest total number of publications in this field, with a notably high proportion of independent research. China ranks second in publication volume, also exhibiting a significant share of independent research, positioning it as one of the core research forces. The volume of publications from countries such as the United Kingdom and Italy decreases successively, exhibiting a “head-heavy” distribution of research output. Concurrently, the red segments for the United States, China, and the United Kingdom are relatively prominent, indicating their status as major participants in multinational collaborative research. For most other nations, the proportion of collaborative research is relatively small. Collectively, this reflects a research landscape characterized by “the United States and China as the core producers of research output, where independent research is the mainstream, and core nations engage in multinational collaboration.”

In Figure 5B, circle size corresponds to a country’s research participation level. The United States, as the largest node, serves as the core leader in research and collaboration within this field. China and the United Kingdom are the next major contributors, while Germany, Italy, and others are active collaborators. The thickness and number of connecting lines represent the intensity of collaboration. The United States, as the “central hub,” engages in high-frequency collaboration with countries such as the UK, China, France, and Germany. China’s collaborations primarily revolve around core partners like the US and the UK. Overall, this field exhibits a “core dominance + regional clusters” collaboration pattern, visually revealing an international cooperation network structure centered on the United States, with China and the UK as key participants and Europe forming regional clusters.

The triple-line association diagram (Fig. 5C) integrates “country-author-keyword” relationships. The left column corresponds to research institute countries/regions, the middle column to core authors, and the right column to research keywords. Connections reveal associations: countries like the UK, Japan, and the US in the left column are linked to core authors such as Inoue Y and Santamaria N. These authors, in turn, connect keywords like “pressure ulcer,” “ wound healing.” The dense connections in the right column also indicate that research themes in this field are relatively focused on PIs, wound healing, and dressing-related technologies.

Table 3 lists the top 5 countries by publication output in the field of dressings and PIs. Scholars from the United States are the most prolific contributors, with 420 publications: the highest number globally. Beyond its leading publication volume, the US also records the highest citation count, which reflects the breadth and depth of its research activities. These achievements highlight the U.\S’s leading role in this research field and have garnered extensive international attention.

**Table 3:** Top 10 countries in the field of pressure wound dressing.

Rank	Country	Documents	Citations	Average Citation/Publication
1	USA	420	11,688	27.83
2	People’s Republic of China	187	3037	16.24
3	England	108	3138	29.06
4	Australia	81	1930	23.83
5	Italy	66	1223	18.53

USA = United States of America.

China follows closely, demonstrating strong research capabilities with a total of 187 publications and 3037 citations. This data indicates China’s active engagement in research on dressings and PIs, with its research outcomes also exerting a certain level of influence in the international academic community.

It is worth noting that the United Kingdom stands out in terms of average citations per paper. UK scholars have published 108 papers, which have accumulated 3138 citations in total, with an average citation rate of 29.06 per paper, these findings highlight the UK’s significant contributions and the high caliber of research in this domain. Its published papers are highly regarded by international peers for their academic value and innovation. Each paper has the potential to spark extensive discussion and in-depth research within the academic community.

## 4. Visualization analysis and discussion

### 4.1. Keyword co-occurrence analysis

**Figure 6.**
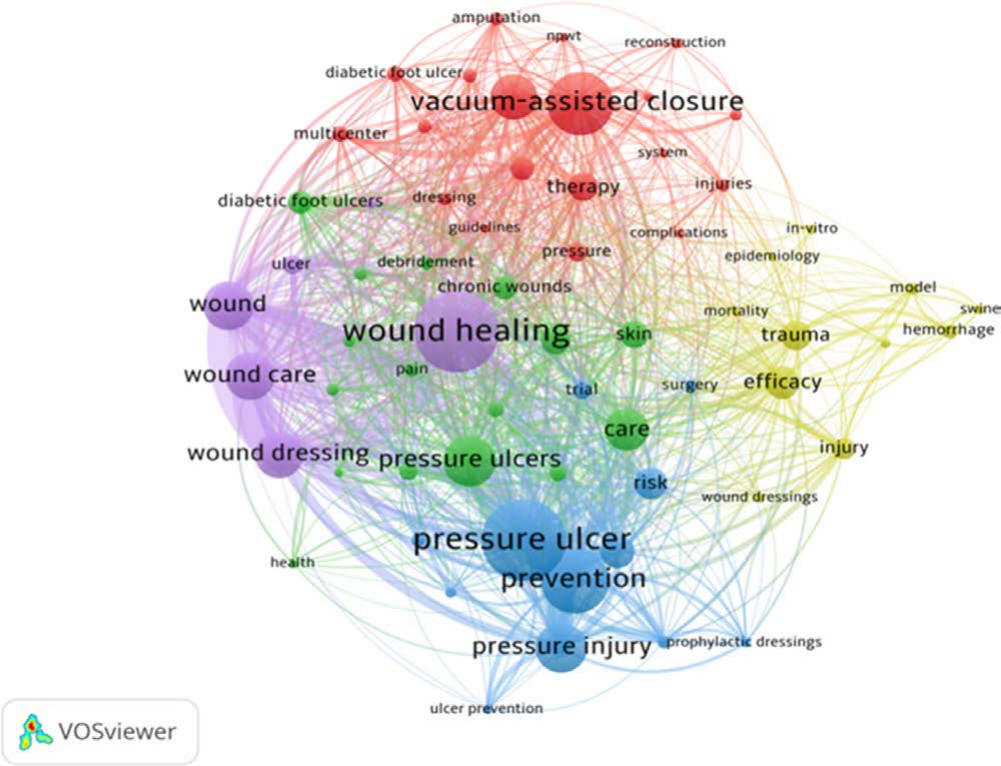
Keyword co-occurrence analysis.

Keywords play a critical role in summarizing the essence and thematic focus of scholarly articles. By employing keyword co-occurrence analysis, researchers can uncover prominent research areas and emerging trends within a given discipline. In the context of this study, which investigates dressings for PIs, we leveraged VOSviewer, a robust bibliometric software, to analyze a dataset of 1253 articles. To ensure the analysis was both focused and meaningful, we applied a frequency threshold of 20 occurrences, resulting in the identification of 67 significant keywords. These keywords were then visualized as a co-occurrence network, illustrated in Figure 6, offering a clear and interpretable overview of the field’s research dynamics.

In Figure 6, larger circular nodes indicate higher keyword frequency, representing key research areas. Wound healing, pressure ulcer, and vacuum-assisted closure (VAC) emerge as the 3 core research topics. Node connections represent association strength, with thicker lines indicating more co-occurrences in the same literature. The strong linkages between negative pressure technology and diabetic foot ulcers, as well as PIs and prophylactic dressings, clearly illustrate the field’s 3 major research pillars (“treatment [negative pressure], care [healing], prevention [dressings]”) and their intersecting thematic logic. Node colors denote distinct clusters: the red cluster centers on negative pressure technology, focusing on treating difficult-to-heal wounds like diabetic foot ulcers; the purple cluster revolves around wound healing, emphasizing chronic wound care and dressing applications; the blue cluster prioritizes pressure injury prevention, linking to research on prophylactic dressings.

To make the analysis results more detailed and in-depth, and to further meet the needs of researchers for deep exploration of key information, we not only relied on the visualization but also conducted more meticulous data organization. Specifically, high-frequency keywords with an occurrence frequency exceeding 80 were extracted separately and compiled into Table 4.

**Table 4:** Lexical prevalence analysis results.

Rank	Keyword	Occurrences	Total link strength
1	pressure ulcer	194	230
2	wound healing	194	215
3	Prevention	157	206
4	vacuum-assisted closure	151	173
5	negative pressure wound therapy	109	144
6	Care	101	147
7	Efficacy	80	102
8	Prevalence	78	115
9	Risk	76	104
10	Trauma	72	70
11	Therapy	69	75
12	Skin	67	85
13	chronic wounds	56	70
14	diabetic foot ulcers	56	89
15	Injury	51	81

Through a comprehensive analysis of Figure 6 and Table 4, we can discern that high-frequency keywords such as “pressure ulcer,” “wound healing,” “prevention,” “VAC,” and “negative pressure wound therapy” have become representative terms in this field. These terms, akin to the cornerstone and pillars of this research domain, collectively reflect the core concerns, main research directions, and potential future trends in the study of dressings for PIs. They provide valuable references and research guidance for subsequent studies.

In recent years, as research has deepened, the selection of dressings for pressure ulcers has shown a significant trend toward functional integration and personalized interventions. For new device-related PIs, particularly in high-risk areas like the face, Biatain silicone foam dressings have demonstrated outstanding advantages in noninvasive ventilated patients due to their unique softness and pressure-dispersing properties. A 2024 randomized controlled trial indicated^[32]^ that compared to traditional ulcer dressings, Biatain foam dressings reduced the incidence of facial PIs by 40%, while improving patient comfort by 35%. This outcome underscores the close association between keywords such as “pressure ulcers,” “prevention,” and “novel dressings,” highlighting the importance of selecting appropriate dressings for preventing PIs in specific scenarios. For deep tissue injuries, the antimicrobial properties of nano-silver ion dressings have been further optimized. A 2021 study confirmed that^[33]^ these dressings deliver greater bactericidal efficacy for diabetic foot ulcers. This research outcome is reflected in the co-occurrence of keywords like “pressure ulcers,” “wound healing,” and “antimicrobial properties,” underscoring the emphasis on and breakthroughs in antimicrobial functionality within dressings to promote wound healing.

The comparative efficacy of dressings guided by the Moist Healing Theory emerged as a research hotspot in 2022. A Bayesian network meta-analysis involving 4444 patients serves as an exemplary study in this field.^[34]^ This study confirmed that silver ion dressings, metallic silver dressings, and hydrogel dressings demonstrate optimal efficacy in pressure injury-related surgical wound healing, prevention of surgical site infections, and reduction of dressing changes, respectively, providing evidence-based support for clinical selection of pressure injury dressings.

The application of VAC systems in traumatic wound repair has emerged as a significant research focus. A study involving 21 patients^[35]^ with postoperative abdominal wound dehiscence demonstrated that the use of a VAC system set to continuous negative pressure ranging from −75 to −125 mm Hg, combined with sharp debridement, effectively promotes abdominal wall wound healing, achieving an overall effective healing rate exceeding 90%.

Whether it be dressings or adjunctive therapeutic technologies, achieving optimal wound healing outcomes requires targeting the intrinsic mechanisms of wound repair, with macrophage phenotype regulation serving as a core therapeutic target, focusing on macrophage phenotype regulation.^[36]^ Strategies such as optimizing material physicochemical properties and loading bioactive molecules promote polarization of pro-inflammatory M1 macrophages toward the reparative M2 phenotype. This balances cytokine levels at the wound site, breaking the chronic inflammation cycle. Simultaneously, it constructs a regenerative microenvironment that stimulates angiogenesis and collagen remodeling, facilitating the transition from the inflammatory phase to the proliferative and remodeling phases of wound healing.

With deepening understanding of wound healing mechanisms and advancements in materials science, research on pressure injury dressings is evolving toward smarter solutions. Ssmart wound dressings are a series of wound dressings that can interact with the wounds,^[37]^sense and react to the wound condition or environment changing by employing built-in sensors and/or smart materials such as stimuli-responsive materials and self-healing materials. To date, different categories of smart wound dressings, such as biomechanical wound dressing, stimuli-responsive wound dressing, self-healing wound dressing for motional wounds, self-removable wound dressing and monitoring wound dressing have been developed. These wound dressings can interact with the wound environment, reflect the changes during the wound healing process, control wound infection, prevent the development to chronic wounds and facilitate autonomous wound healing.

### 4.2. Keyword evolution analysis

**Figure 7.**
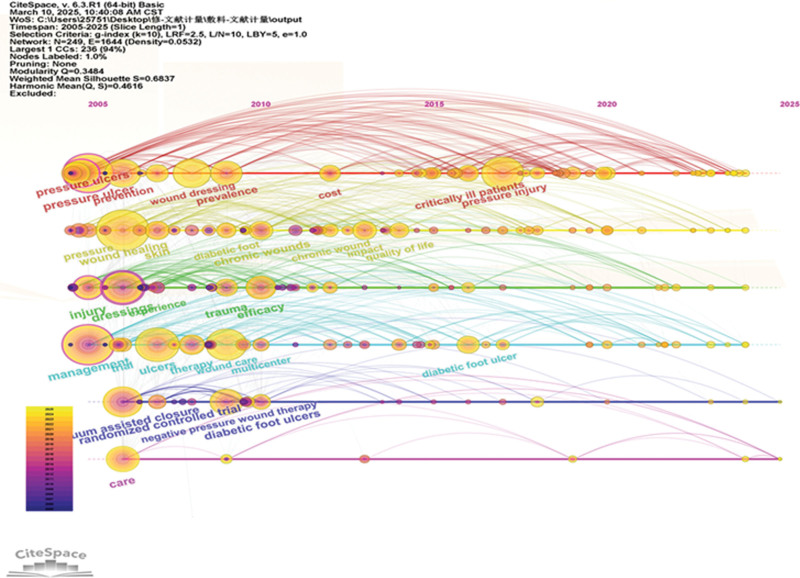
Keyword timeline map from 2005 to 2025.

Keywords serve as concise representations of research content. Although keyword co-occurrence analysis can effectively reveal the background, hotspots, and focal points within a research domain, conducting evolutionary analysis on keywords helps achieve an in-depth understanding of its developmental path. Given this context, we employed CiteSpace to conduct an evolutionary analysis on keywords extracted from literature related to dressings for PIs, visualizing the results through a timeline mapping (Fig. 7).

The timeline graph in Figure 7 presents a wealth of information. The horizontal axis represents the period from 2005 to 2025, clearly illustrating the progression of research over time. The vertical axis features cluster labels in different colors, representing distinct research theme clusters. In the timeline graph, each horizontal line corresponds to a cluster, and the nodes on the line represent keywords within that cluster. Node dimensions mirror the keyword’s occurrence rate in the corresponding year, with larger dimensions signaling more focus on the keyword that year.

From the graph, it is evident that “pressure injury” and “wound care” as significant cluster themes have consistently garnered attention throughout the period, indicating their status as core research topics in this field. The varying node sizes of keywords such as “pressure ulcer” and “wound dressings” across different years reflect the dynamic evolution of research interest. The connecting lines between clusters illustrate the associations and interactions of keywords across different research themes. Some lines become denser in specific periods, indicating closer connections between research themes during those times, possibly suggesting cross-disciplinary integration. For example, the connecting line between the cluster of “diabetic foot ulcers” and the “wound dressing” cluster reflects ongoing research exploration in the application of dressings for diabetic foot ulcers, with mutual influence between different research themes. By analyzing the timeline graph in Figure 7, we can systematically trace the developmental trajectory of research on dressings for PIs, and clarify the evolution of various research themes across different periods, and their interrelationships, providing comprehensive and valuable references for further in-depth research.

Keywords, as condensed expressions of research cores, reveal the evolutionary trajectory of disciplinary hotspots and developmental trends. The analysis demonstrates that 1 prominent current research hotspot centers on smart wound dressings innovation. The convergence of materials science and biomedical engineering has catalyzed the emergence of intelligent dressings. These advanced systems autonomously modulate their performance in response to wound microenvironmental variations (e.g., pH, temperature, humidity, inflammatory cytokine concentrations), enabling targeted drug release and adaptive wound healing regulation.

### 4.3. Burst term analysis

**Figure 8.**
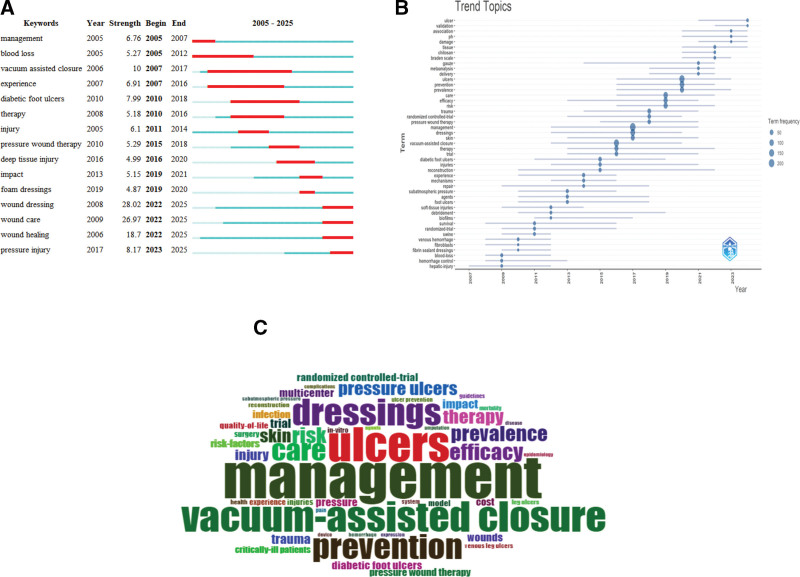
(A) The top 15 keywords exhibit the most significant citation bursts, (B) trend plots of dressings and stress injuries, (C) keyword cloud of the top 50 keywords.

Analysis of 2005 to 2025 data using VOSviewer and Bibliometric revealed multiple keywords with significant attention surges in specific years, indicating intensified research focus in related domains (Fig. 8A).

The emergence of “management” and “injury” in 2005 corresponded to the initial standardization of wound management systems. The rising prominence of “diabetic foot ulcers” in 2010 aligned with updated clinical guidelines and heightened clinical attention to diabetic foot care. Post-2017, the increased focus on “pressure injury” and “deep tissue injury” reflected revisions to pressure injury staging criteria and the widespread adoption of deep tissue assessment techniques. The sustained interest in “wound dressing” and “wound healing” throughout 2022 underscored the ongoing development of advanced wound care technologies. “Deep tissue injury” reflected revisions to pressure injury staging criteria and the widespread adoption of deep tissue assessment techniques. The sustained prominence of ”wound dressing“ and ”wound healing” in 2022 mirrors the surge in research on novel functional wound dressings and the academic focus on healing mechanisms. These temporal fluctuations in search volume fundamentally reflect the interplay of field demands, technological breakthroughs, and academic trends.

The trend chart (Fig. 8B) illustrates temporal research intensity variations. Terms like “ulcer,” “validation,” and “association” showed increased frequency post-2017, suggesting an enhanced focus on ulcer pathophysiology validation and comorbidity studies. Concurrently, high-frequency terms such as “management,” “care,” and “efficacy” reflect the prioritization of therapeutic outcomes and care quality.

The word cloud (Fig. 8C) highlights dominant terms including “ulcers,” “management,” “dressings,” “VAC,” and “prevention,” collectively emphasizing scholarly prioritization of comprehensive wound care strategies encompassing therapeutic interventions and propylactic approaches.

### 4.4. Co-citation analysis

**Figure 9.**
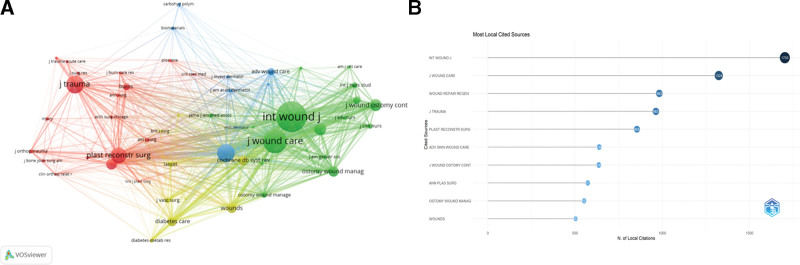
(A) Publications with references (generated byVOSviewer software), (B) total Number of publications of top 10 references (generated by Bibliometrix software).

Co-citation analysis seeks to identify frequently cited papers and their associated journals within a research field. Employing VOSviewer and Bibliometric for journal co-citation mapping, we established a minimum citation threshold of 100, leading to the selection of 55 journals for analysis. The resulting co-citation network is illustrated in Figure 9.

Figure 9 shows that the journal citation network comprises 4 distinct clusters, each represented by a unique color. The top 3 most cited journals are the Journal of Wound Care (1327 citations), Wound Repair and Regeneration (983 citations), and International Wound Journal (1708 citations). All these journals belong to journal citation reports Category 1.

Within the 4-category classification system derived from cluster analysis, publications assigned to the Azure group exhibited cluster concentration on basic research and clinical practice regarding pressure injury treatment. Centered around the International Wound Journal, studies employ cellular experiments and animal models to investigate mechanisms of dressings, such as the antibacterial properties and cellular interactions of nano-silver dressings. Journals like the Journal of Wound Care utilize clinical trials to document healing outcomes of various dressings for PIs, providing evidence-based guidance for clinical selection. The red cluster journals emphasize interdisciplinary innovation. Materials science journals employ 3D printing and electrospinning to develop advanced dressings (e.g., smart hydrogel dressings), while biomedical engineering journals pioneer wearable wound monitoring devices: these devices collect real-time wound data that can be processed by AI algorithms for dynamic dressing optimization. This cross-domain integration creates technical conditions for developing AI-assisted dressing selection systems.

Analysis of the top 10 most cited papers (2005–2025) in pressure injury dressings using VOSviewer (Table 5) elucidates the research trajectory of this field.

**Table 5:** Important papers in the field of pressure injury dressings.

Rank	cited reference	citations	total link strength
1	Morykwas MJ, 1997, Ann Plast Surg	104	121
2	Argenta LC, 1997, Ann Plats Surg	91	109
3	Santamaria N, 2015, Int Wound J	76	137
4	Armstrong DG, 2005, Lancet	73	99
5	Blume PA, 2008, Diabetes Care	58	81
6	Levy A, 2015, J Tissue Viability	47	115
7	Call E, 2015, Int Wound J	42	89
8	Kalowes P, 2016, Am J Crit Care	42	94
9	Levy A, 2016, Ostomy Wound Manag	31	93
10	Santamaria N, 2015, J Wound Care	30	94

In terms of citation counts, Morykwas MJ and Argenta LC 1997 paper in Annals of Plastic Surgery (104 citations) and Argenta LC work (91 citations) rank highest, demonstrating their foundational contributions to the field. In contrast, Levy A 2016 paper in Ostomy Wound Management (31 citations), though less cited currently, may indicate emerging research directions due to its recent publication. Multiple highly cited papers were published in specialized journals like Annals of Plastic Surgery and International Wound Journal, highlighting their disciplinary authority and the interdisciplinary connections between wound care, plastic surgery, and pressure injury research. Regarding authorship, Santamaria N and Levy A, each contributed 2 papers, solidifying their roles as leading researchers driving high-impact studies in this domain. The “total link strength” metric reveals that Santamaria N 2015 paper in International Wound Journal (link strength: 137) occupies a central position in the research network, strongly connecting diverse studies. Collectively, these highly cited papers provide critical insights through foundational contributions, author leadership, journal dissemination, and knowledge network construction, offering valuable guidance for future research.

## 5. Conclusions and prospects

### 5.1. Conclusions

As an interdisciplinary field integrating medicine, materials science, and nursing, pressure injury dressing research has grown sustainably driven by clinical demand and technological advancements. Bibliometric analysis (VOSviewer, Bibliometric) of 2005 to 2025 literature clarifies its core characteristics:

1. Core Research Communities and Collaboration: influential scholars (e.g., Santamaria N, Levy A) lead research on dressing innovation and pathological mechanisms, with expanding cross-institutional and international collaborations, though co-citation networks show discrete clusters and untapped cross-domain collaboration potential;
2. Scholarly Communication: high-impact journal citation reports Q1 journals serve as core knowledge dissemination platforms, with open-access publications boosting academic exchange, while citation links between materials science and clinical nursing journals remain weak;
3. Research Trends and Hotspots: keyword and burst term analysis reveals a shift from traditional dressing property evaluation to multifunctional design, marked by time-specific research focuses (e.g., “diabetic foot ulcers” in 2010, “pressure injury” in 2017, sustained “wound dressing” post-2022), current hotspots in antimicrobial mechanisms and vulnerable population interventions, and emerging frontiers like 3D-printed dressings driven by interdisciplinary integration;
4. Core Knowledge Framework: co-citation analysis identifies foundational literature on pathogenesis, dressing design, and clinical efficacy, forming a core knowledge scaffold, yet gaps exist as foundational studies focus on macro-functionality with limited exploration of dressing-wound microenvironment micro-mechanisms.

### 5.2. Limitations and prospects

This study systematically analyzed the knowledge landscape of pressure injury dressing research via bibliometric methods, but several limitations should be acknowledged. Data source bias: the analysis relies on a single database, which may exclude literature from regional or discipline-specific repositories, potentially narrowing the scope of captured research outputs. Interpretation subjectivity: while bibliometric tools (VOSviewer, Bibliometric) provide quantitative clustering and term analysis, the interpretation of cluster themes and emergent keywords inevitably involves subjective judgment, which may affect the precision of trend delineation. Temporal and thematic coverage: the analysis focuses on 2005 to 2025, but early foundational literature (pre-2005) was not included; additionally, non-keyword dimensions (e.g., funding sources, clinical trial phases) were not explored, limiting the comprehensiveness of the research context.

Looking ahead, several specific research gaps and innovative directions warrant urgent exploration in this field. First, accelerate clinical translation of smart wound dressings systems. Burst term analysis demonstrates that while “wound dressing” remained a sustained high-frequency research focus post-2022, keywords associated with the clinical translation of smart wound dressings (e.g., “sensor dressing clinical trial,” smart wound dressings safety validation,” “translational pathway for advanced dressings”) failed to emerge as burst terms. This finding indicates a notable underdevelopment in research efforts dedicated to translating materials science innovations into clinical practice for pressure injury management. Co-citation cluster analysis reveals a weak knowledge link between the red cluster and the azure cluster. This disconnect suggests that current smart wound dressings research lacks systematic clinical validation: preclinical data are fragmented, and standardized frameworks for efficacy/safety assessment are absent. The future direction is to establish interdisciplinary collaboration platforms integrating materials science, flexible electronics, and clinical nursing; design multicenter, prospective clinical trials to validate the long-term efficacy of smart wound dressings in real-world wound microenvironments.

Second, elucidate the micro-mechanisms of dressing-wound microenvironment interactions. Keyword word cloud and trend analysis show that “efficacy” and “management” are core terms, but terms related to micro-mechanistic exploration are underrepresented. Analysis of highly cited papers indicates that foundational works (e.g., Morykwas MJ 1997 study) focus on macro-functional evaluation of dressings, while few high-impact papers investigate molecular-level interactions between dressings, wound tissue, and microbiota. The future direction is to integrate multi-omics technologies to characterize the tripartite (dressing-host-microorganism) interaction mechanisms in specific wound types; build mechanistic models to guide personalized dressing selection, bridging the gap between macro-clinical outcomes and micro-biological processes.

Third, strengthen cross-cluster knowledge integration. Co-citation network analysis identifies 4 discrete clusters (basic research, clinical practice, interdisciplinary innovation, materials engineering) with limited inter-cluster citation links. For example, materials science-focused journals (red cluster) rarely cite clinical practice journals (azure cluster), and vice versa. The future direction is to promote cross-cluster academic collaboration; encourage high-impact journals to publish interdisciplinary review articles that synthesize knowledge across clusters, to accelerate knowledge translation between innovation and practice.

In conclusion, future research on pressure injury dressings should leverage the bibliometric insights from this study to move beyond traditional material property comparisons: focus on closing the gap between smart wound dressings innovation and clinical translation, deepen mechanistic exploration of dressing-wound interactions, and integrate cross-cluster knowledge: ultimately advancing precision, intelligence, and standardization in pressure injury dressing care.

## Acknowledgments

We convey our appreciation to all the authors who have made contributions to this study.

## Author contributions

**Conceptualization:** Chenxin Zhang.

**Data curation:** Chenxin Zhang.

**Formal analysis:** Chenxin Zhang, Liping Zhang.

**Funding acquisition:** Zhenghui Dong.

**Investigation:** Chenxin Zhang, Liping Zhang.

**Methodology:** Chenxin Zhang, Yi Liu.

**Project administration:** Zhenghui Dong.

**Resources:** Chenxin Zhang, Zhenghui Dong.

**Software:** Chenxin Zhang.

**Supervision:** Yi Liu, Zhenghui Dong.

**Validation:** Chenxin Zhang, Xian Ma.

**Visualization:** Chenxin Zhang, Xian Ma.

**Writing – original draft:** Chenxin Zhang.

**Writing – review & editing:** Chenxin Zhang, Zhenghui Dong.
